# Potential Use of *Torulaspora delbrueckii* As a New Source of Mannoproteins of Oenological Interest

**DOI:** 10.1021/acs.jafc.4c01001

**Published:** 2024-05-09

**Authors:** María Oyón-Ardoiz, Elvira Manjón, María Teresa Escribano-Bailón, Ignacio García-Estévez

**Affiliations:** Department of Analytical Chemistry, Nutrition and Food Science, Universidad de Salamanca, Salamanca E37007, Spain

**Keywords:** mannoproteins, red wine, astringency, color, non-*Saccharomyces* yeast

## Abstract

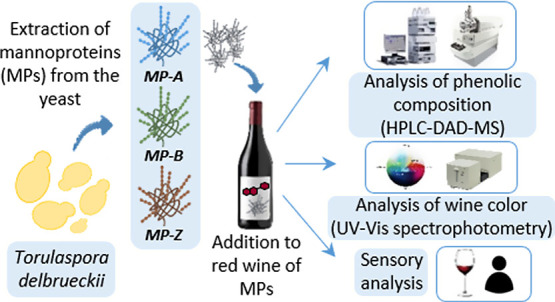

In this work, three MP extracts obtained from *Torulaspora delbrueckii* were added to red wine, and the changes in phenolic composition, color, and astringency were evaluated by HPLC–DAD–ESI–MS, tristimulus colorimetry, and sensory analysis, respectively. The MP extracts modified wine phenolic composition differently depending on the type of MP. Moreover, two MP extracts were able to reduce wine astringency. The fact that the MP-treated wines showed an increased flavanol content suggests the formation of MP-flavanol aggregates that remain in solution. Furthermore, the formation of these aggregates may hinder the interaction of flavanols with salivary proteins in the mouth. The effect of these MPs might be associated with their larger size, which could influence their ability to bind flavanols and salivary proteins. However, one of the astringent-modulating MPs also produced a loss of color, highlighting the importance of assessing the overall impact of MPs on the organoleptic properties of wine.

## Introduction

Phenolic compounds are a type of secondary metabolite derived from plants. In red winemaking, grape solids are kept in contact with the fermenting must allowing the extraction to the wine of several classes of phenolic compounds (i.e., anthocyanins, flavanols, flavonols, and phenolic acids). These compounds are highly related to wine quality since they contribute to wine sensory properties, like color and astringency. In this way, anthocyanins are the main compounds responsible for the color of red wine. These pigments can associate through noncovalent forces with other wine compounds (named copigments) giving place to the phenomenon of copigmentation, which results in a stabilization of the red color of the flavylium form.^1^ Flavanols, flavonols, and phenolic acids can act as copigments, thus contributing to wine color.^2^ Moreover, flavanols and phenolic acids, among other wine compounds, can react with anthocyanins to form anthocyanin-derived pigments.^3^ Flavonols and flavanols can also directly contribute to wine taste and mouthfeel since some studies have related these compounds to wine bitterness and astringency.^4,5^ Astringency can be defined as the tactile sensation of dryness and roughness in the mouth and is mainly produced by wine flavanols. Despite the molecular mechanisms of astringency development are not yet fully characterized, flavanol-salivary protein interaction and/or precipitation is generally the most accepted mechanism.^4^

Over the last years, climate change has had an impact on grapevine phenological development, which can lead to an alteration of the composition of grapes and wines.^6^ Under conditions of increased temperatures, the synthesis of sugars and the degradation of malic acid in grapes occur faster.^7^ This earlier technological maturity of grapes is leading to an advancement in harvest dates.^6^ However, an earlier harvest of grapes implies inadequate phenolic maturity, leading to some sensory alterations, such as an unbalanced wine astringency and a poor or unstable color. In fact, some authors have reported that increased temperatures can cause a decoupling in the accumulation of sugars and anthocyanins in grapes resulting in an imbalance of these compounds.^8,9^ As grape ripens, the possibility of flavanol extraction from grape seeds to the wine decreases due to the continual decrease in the content of flavanols in grape seeds from veraison to harvest and/or to their lesser extractability.^10^ In grapes, procyanidins (PCs) are located in seeds and skins, while prodelphinidins (PDs) are exclusively located in skins. According to sensory analysis, catechins and PCs are more astringent, dry, rough, unripe, and persistent than gallocatechins and PDs, which are smoother, more velvety, and viscous.^4^ Therefore, the earlier harvest of grapes results in higher extraction to the wine of grape seed tannins and consequently in an unpleasant astringency characteristic of these compounds.

In view of this, several viticultural and oenological practices have been proposed to mitigate the negative effects of climate change on wine quality. Among these, the use of mannoproteins (MPs) could help stabilize wine color and reduce wine astringency.^11−15^ MPs are glycoproteins located in the yeast cell wall that are characterized for being heavily glycosylated with mannose residues.^16^ In wine, MPs are naturally released from *Saccharomyces cerevisiae* during the different winemaking stages.^17^ However, not all the MPs exert the same effects in wine and some controversies regarding their potential to modulate color and astringency have been reported in the literature.^18−22^ These different effects of MPs are most likely due to the high structural and compositional heterogeneity of this class of biopolymers.^11,13,18^ Unveiling the relations between structure/composition of MPs and their techno-functional properties in wine is needed since it would allow the use of MPs as an efficient tool to counteract some climate change consequences in the winemaking industry.

The use of non-*Saccharomyces* yeast in oenology is currently under research as they can produce wines with lower ethanol content, higher aroma complexity, improved mouthfeel, etc.^23^ In previous studies carried out in our laboratory, we have explored the potential of MPs derived from different yeast species to modulate wine sensory properties.^13,24^ In one of these studies, it has been demonstrated the ability of MPs obtained from an oenological strain of *Torulaspora delbrueckii* to interact with grape seed tannins, which could indicate the potential of these MPs to modulate wine astringency.^24^ Therefore, the objective of this work is to deepen the possibilities of the use of *T. delbrueckii* MPs for the modulation of wine organoleptic properties. In this sense, the effect of different MP extracts obtained from *T. delbrueckii* on red wine phenolic composition, color, and astringency has been evaluated.

## Materials and Methods

### Extraction and Characterization of MPs from *T. delbrueckii*

Three MP extracts were obtained from a commercial strain of *T. delbrueckii* (BIODIVA, Lallemand Inc., Montreal, Canada) according to the procedure previously developed in our laboratory.^24^ Briefly, the yeast was grown in liquid yeast extract peptone dextrose (YPD) until OD_600 nm_ 14–16. The obtained biomass was subjected to three different treatments for the extraction of MPs. First, yeast biomass was submitted to an induced autolysis in NaCl 3% (to obtain the MP-A extract). Then, autolyzed cells were collected by centrifugation, and an aliquot was subjected to enzymatic hydrolysis (to obtain the MP-Z extract) with a β-glucanase (Zymolyase 20T, US Biological, Salem, MA, USA) and another aliquot was subjected to chemical hydrolysis with a base (to obtain the MP-B extract). Total protein content, molecular weight (MW) distribution, and monosaccharide composition of the MP extracts were determined according to Oyón-Ardoiz et al.^24^

### MP Addition to Red Wine

A Tempranillo red wine (D.O. Toro, Valladolid, Spain) was selected because of its intense astringency. Wine samples were taken from the barrel after 2 months of aging with no prior stabilization treatments conducted. Previous to the addition of MPs, the wine was centrifuged (1030 g, 10 min) to avoid the presence of particles in suspension. Then the three MP extracts were added to the wine at a concentration of 400 mg/L, with this dose being the maximum permitted by the European Community (EC Regulation No. 606/2009). This resulted in four wine samples named *A* wine, *Z* wine, and *B* wine (for the samples supplemented with MP-A, MP-Z, and MP-B extracts, respectively) and the control wine (not supplemented wine). All wine samples were prepared in triplicate and stored at room temperature in darkness for 7 days (sampling point P0). Then, they were subjected to a cold stabilization treatment to provoke a colloidal destabilization, consisting of cooling the wine to approximately 0 °C in darkness for 7 days (sampling point P1). Afterward, wine samples were stored at cellar temperature (∼15 °C) and darkness for 45 days (sampling point P2) to evaluate the effect of MPs on the evolution of the wine phenolic profile. In these three sampling points (P0, P1, and P2), an aliquot of each sample was taken and centrifuged (3590 g, 10 min). The color of the resulting supernatant was evaluated by tristimulus colorimetry, and the detailed phenolic composition was analyzed by HPLC–DAD–ESI–MS.

### HPLC–DAD–ESI–MS Analyses

The analysis of anthocyanins, derivative pigments, and flavonols was carried out by using an Agilent 1100 series HPLC (Agilent Technologies, Waldbronn, Germany) equipped with a C18 reversed-phase column (5 μm, 150 mm × 4.6 mm) (Aqua, Phenomenex, Torrance, CA) thermostated at 35 °C. The conditions of the chromatographic analysis used in this study were previously developed in our laboratory for the analysis of wine samples.^25^ Identification of chromatographic peaks was carried out by coupling a 3200 Qtrap (Applied Biosystems) mass spectrometer. The conditions of the mass spectrometer employed in this study are described in detail by Alcalde-Eon et al.^12^ For the quantification of wine pigments and flavonols, chromatograms were registered at 520 and 360 nm, and malvidin-*O*-glucoside and quercetin-3-*O*-glucoside calibration curves were employed, respectively.

Prior to the HPLC–DAD–ESI–MS analysis of flavanols and phenolic acids, anthocyanins were removed from wine samples using a cationic exchange cartridge (Oasis MCX, Waters Corp., Milford, MA, USA) according to the method described by García-Estévez et al.^26^ Before HPLC–MS analysis, chlorogenic acid was added to the samples as an internal standard (final concentration of 0.025 mg/mL). Chromatographic analyses were performed using an Agilent 1200 series HPLC instrument (Agilent Technologies, Waldbronn, Germany) equipped with an Agilent Poroshell 120 EC-18 column (2.7 μm, 4.6 mm × 150 mm) (Agilent Technologies, Waldbronn, Germany) thermostated at 25 °C. Quantification of flavanols was carried out by mass spectrometry using the same equipment as described above. HPLC and mass spectrometer conditions are described in detail by García-Estévez et al.^26^ Calibration curves of (+)-catequin, (−)-epicatequin, PC dimers B1 and B2, PC trimer C1, (−)-epicatequin 3-*O*-gallate, (+)-gallocatequin and (−)-epigalocatequin were employed. For the quantification of phenolic acids, chromatograms were registered at a preferred wavelength of 330 nm, and calibration curves of caffeic, *p*-coumaric, ferulic, and gallic acids were employed.

### Colorimetric Measurements

After centrifugation of wine samples, an aliquot of the supernatant was filtered using a 0.22 μm filter to avoid the presence of particles in suspension. The absorption spectra (190–770 nm) were registered in a Hewlett-Packard 8453 UV–vis spectrophotometer (Agilent Technologies, Waldbronn, Germany) employing quartz cuvettes of 1 mm path length. Synthetic wine (5 g/L tartaric acid, 12% (*v/v*) ethanol, 11.65 g/L NaCl, pH 3.6) was employed as blank. From the visible spectra (380–770 nm), the CIELAB parameters (L*, a*, and b*) were calculated with the software CromaLab^27^ using the CIE standard illuminant D65 and the CIE 1964 standard observer as references. From the obtained color coordinates a* and b*, the chroma or saturation of the color (C*_ab_) and the hue (*h*_*ab*_) were calculated. Color differences (ΔE*_ab_) between the wine samples supplemented with MPs and the control wine were calculated according to the following formula:
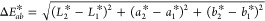


### Sensory Analysis

Astringency intensity was evaluated by a panel composed of 8 panelists (2 men and 6 women) aged 25–55 years old. All panelists were previously trained to recognize and rate the intensity of astringency by using, as a training standard, grape seed extract. The grape seed extract was obtained according to the method described by Oyón-Ardoiz et al.^24^ The training was carried out in two sessions. In each session, a triangle test with two concentrations of the extract (1 and 1.5 mg/mL) and a sorting task with concentrations ranging from 0 to 1.5 mg/mL were performed. The panelists were also asked to rate the perceived astringency intensity in a Labeled Magnitude Scale (LMS) in order to become familiar with this form of rating.

In sampling point P2 (after storage of the wine for 45 days), the astringency intensity of the wine samples was rated using the LMS scale. To avoid any bias, samples were presented to the panelists in a randomized way in covered drinking containers labeled with three-digit random numbers. Panelists took 8 mL of each sample, tasted it, and spitted out without knowing the nature of the sample.

### Statistical Analyses

Statistical analyses were performed using the software IBM-SPSS Statistics v. 28. The statistical significance of the differences between the control wine and the wines supplemented with MPs were calculated by a Student’s *t*-test. Regarding wine color and phenolic composition, differences among MP-added samples were determined by a one-way analysis of variance (ANOVA) followed by a posthoc Tukey-b test. In all cases, differences were considered significant when *p* < 0.05.

## Results and Discussion

### MP Extract Characterization

The characterization of the MPs revealed important structural and compositional differences among them. Specifically, the MW distribution of the extracts (Table S1 in the Supporting Information) exhibited significant differences. MP-A contained the largest MPs (average MW of ∼129 kDa), followed by MP-Z (∼116 kDa) and MP-B (∼94 kDa). Likewise, the monosaccharide composition of the MP extracts (Table S2 in the Supporting Information) also showed substantial variations. It is noteworthy that mannose predominated in all three extracts, indicating the release of MPs by the treatments applied to *T. delbrueckii*. A higher content of glucose was observed in MP-Z, suggesting the release of portions of cell wall β-glucan or fragments composed by a MP connected to a portion of β-glucan through the enzymatic treatment. Regarding MP-A, yeast cell autolysis resulted in an important content of ribose, which could be explained by the release of nucleic acids during the autolytic process. As for the protein content (Table S1 in the Supporting Information), the three MP extracts showed low protein percentages (3–4%), with no significant differences among them.

### Evaluation of the Changes in Phenolic Composition after MP Addition

Cold stabilization of wine is a common method used in wineries to precipitate unstable tartrate salts and colloidal matter, preventing the formation of haze and sediments once the wine is bottled. It consists of cooling down the wine to temperatures close to the freezing point for a few days, inducing a colloidal destabilization and the crystallization of tartrate salts.^28,29^ The addition of MPs could enhance colloidal stability since MPs, as well as other wine polysaccharides, are shown to act as colloidal stabilizers. However, the role of MPs in wine colloidal state is not well-known, and controversial results have been reported. In this sense, some studies have shown that MPs could prevent the aggregation of wine polyphenols. For example, Nguela et al. found that the interaction between MPs and polyphenols resulted in stable colloidal dispersions.^30^ In addition, some studies have described the stabilization of tannin particles by MPs.^19,31,32^ On the contrary, Guadalupe and co-workers reported that the use of MPs and of MP-overproducing strains conduced to wines with lower polyphenol content, suggesting the precipitation of MP-polyphenol aggregates.^21,22^

With the purpose of evaluating the effect of the addition of the obtained MPs on the stability of wine colloids and on wine phenolic composition, wine samples were stored at room temperature and darkness for 7 days (sampling point P0), and then, samples were maintained at 0 °C and darkness for 7 days more (sampling point P1). After the wines were subjected to the cold stabilization treatment, wine samples were stored at cellar temperature and darkness for 45 days (sampling point P2) in order to analyze the effect of MP supplementation in the evolution of wine phenolic composition. In these three sampling points, the wine phenolic composition was analyzed by HPLC–DAD–ESI–MS.

#### Phenolic Acid Content

**Figure 1:**
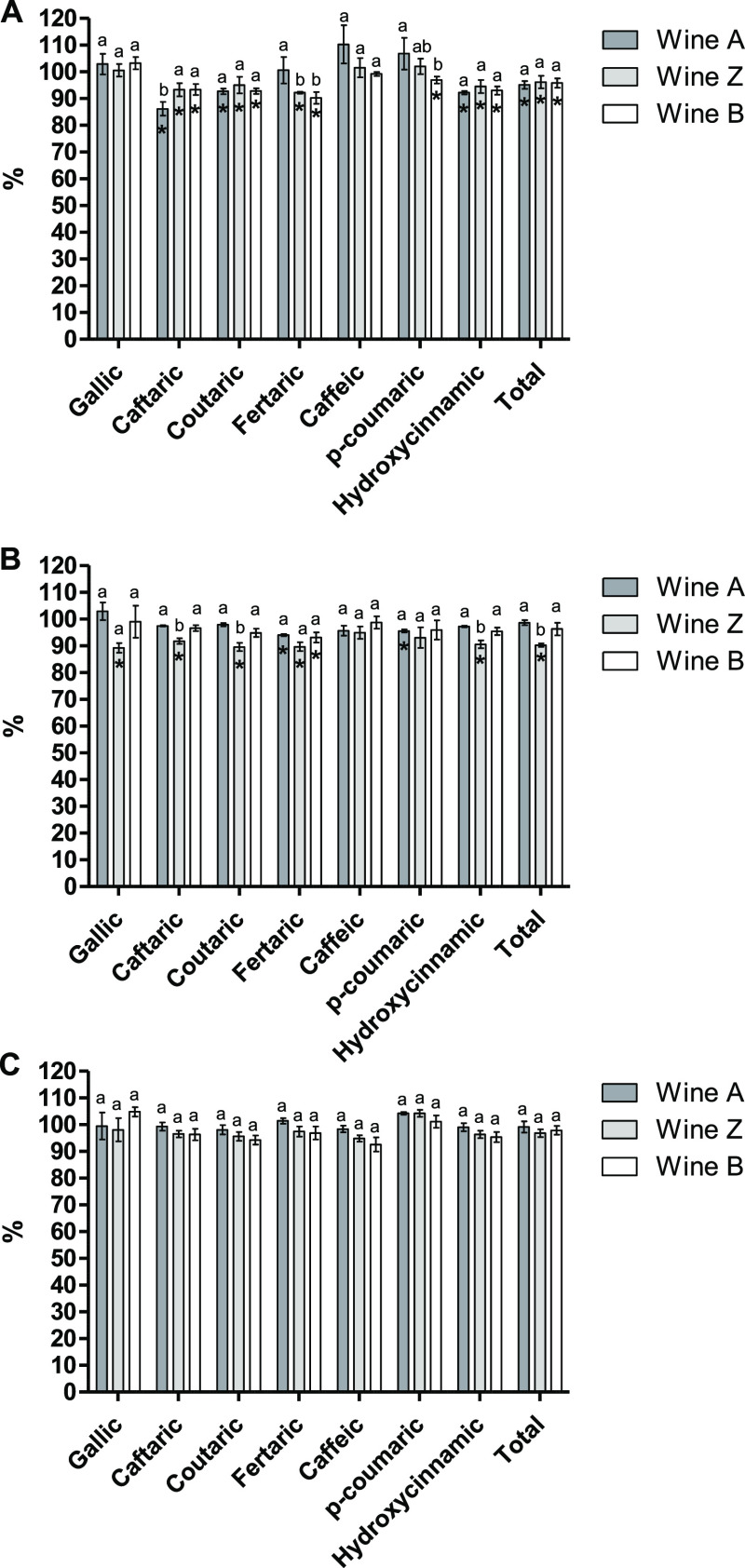
Percentage (%) of phenolic acids with respect to the control wine at P0 (A), P1 (B), and P2 (C) sampling points. Different letters indicate significant differences among MP supplemented wines (*p* < 0.05). Significant differences among the control wine and the wines added with MP are indicated with an asterisk (*p* < 0.05).

HPLC–DAD–ESI–MS analyses allowed the identification of 8 phenolic acids (see Table S3 in the Supporting Information): gallic, *cis*-caftaric, *trans*-caftaric, *cis*-coutaric, *trans*-coutaric, *trans*-fertaric, caffeic, and *p*-coumaric acids. The sum of hydroxycinnamic acids (HCAs) represented ∼73% of the total content of phenolic acids, while gallic acid, as the only hydroxybenzoic acid (HBA) identified in this wine, accounted for ∼27%. In P0, a significant decrease of the tartaric esters of HCAs in the wines enriched with MPs was observed in comparison to the control wine (see caftaric, coutaric, and fertaric columns in Figure 1A). Since esterified HCAs were the most abundant phenolic acids in this wine (representing ∼69% of the total), the decrease in their content led to a slight (∼5%) but significant decrease in the total content of phenolic acids in P0. Guadalupe and Ayestarán observed a decrease in the esterified HCAs during the malolactic fermentation of wines enriched with commercial MPs that was accompanied by an increase in the free forms.^21^ Therefore, these authors proposed that hydrolysis of the tartaric esters was promoted in the MP-enriched wines. However, this does not seem to be occurring in our study, as the decrease of the esterified HCAs was not accompanied by a significant increase in their respective free forms (i.e., caffeic and *p*-coumaric acids). Consequently, this could be attributed to an enhanced precipitation of the esterified forms of HCAs in the presence of the MPs. Moreover, considering that the decrease in the HCAs content mainly affected the esterified forms compared to the free acids, it suggests that the esterification of the HCAs may facilitate their interaction with MPs and/or the formation of unstable colloids that would subsequently precipitate.

After the cold stabilization treatment (P1), significantly lower levels of esterified HCAs were found in *Z* wine compared to the control wine, which translates into a significantly lower content of total HCAs (Figure 1B). However, in *A* and *B* wines, the decrease was significant only for the fertaric acid content. The changes produced by the MPs addition seem to decrease over storage time since in P2, no significant changes can be observed between the MP-supplemented wines and the control wine (Figure 1C). This is in agreement with Guadalupe and co-workers, who found that the use of a MP overproducing yeast strain and the use of a commercial-rich MP preparation had no impact on the content of HCAs.^20^ However, Sartor et al. found that the concentrations of caffeic, *p*-coumaric, and caftaric acids in rosé sparkling wines treated with a commercial MP were higher than in the control wine (untreated wine) after 12 months of on-lees aging.^33^ del Barrio-Galán et al. showed variable effects of different yeast derivative products in the content of HCA tartaric esters of red and white wines.^18^

Finally, regarding HBAs, the content of gallic acid seemed to be almost unaffected by the addition of the MPs since, with the exception of *Z* wine in P1, no significant differences were observed among the MP-treated wines and the control wine at the three analyzed sampling points (Figure 1A–C). This contrasts with the findings of Sartor et al., who reported higher HBA content in sparkling wines added with a commercial MP after 12 months of aging compared to untreated wines.^33^ On the contrary, del Barrio-Galán and co-workers observed a decrease in the content of HBAs in red wines treated with a commercial inactive dry yeast product rich in low MW MPs after 2 and 4 months of treatment.^34^ Therefore, the different results reported in the literature regarding the effects of MPs on the composition of phenolic acids, as well as those found in this study, could arise from the different characteristics of the MPs used.

#### Flavonol Content

**Figure 2:**
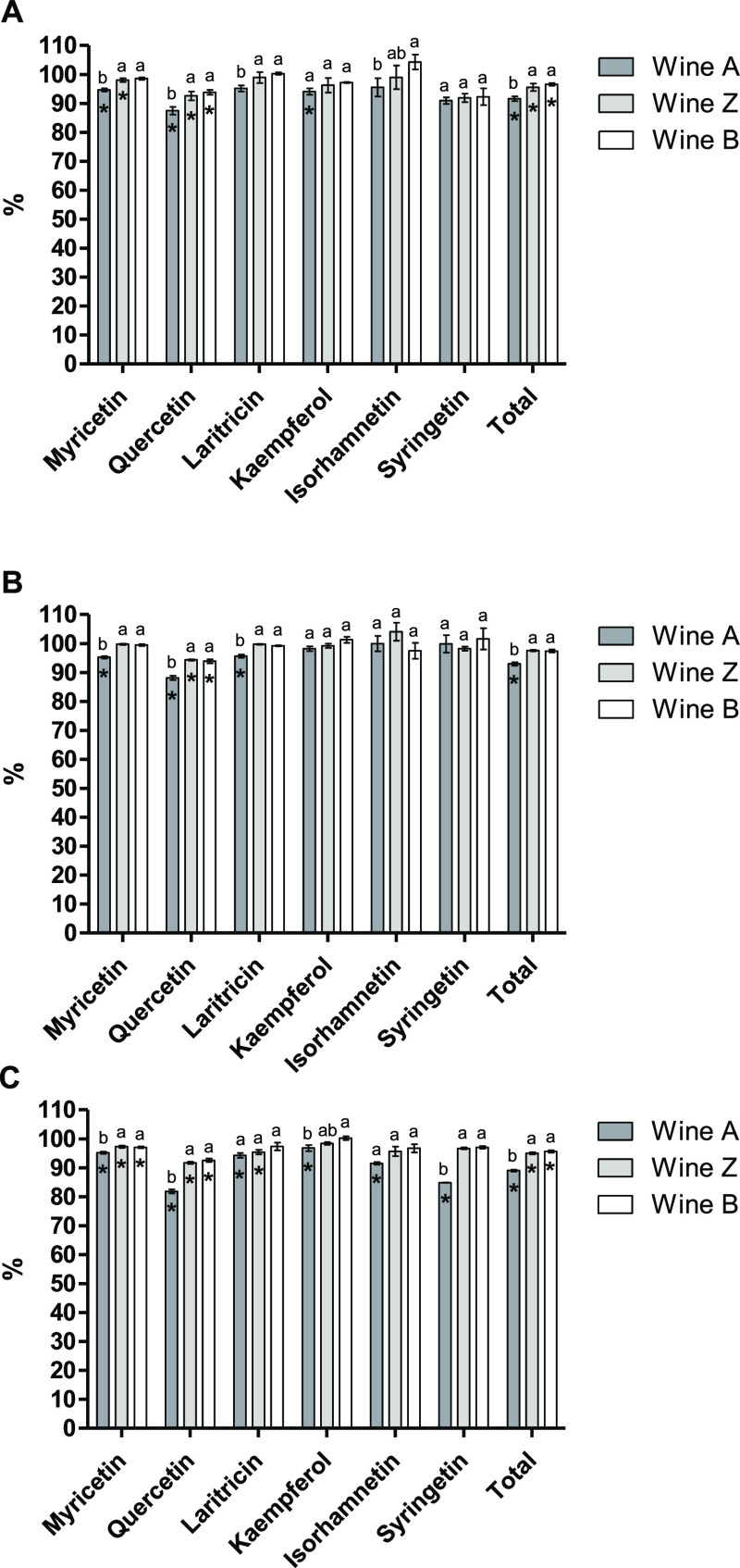
Percentage (%) of flavonols with respect to the control wine at P0 (A), P1 (B), and P2 (C) sampling points. Different letters indicate significant differences among MP supplemented wines (*p* < 0.05). Significant differences among the control wine and the wines added with MP are indicated with an asterisk (*p* < 0.05).

Thirteen flavonols were identified by HPLC–DAD–ESI–MS (see Table S4 in the Supporting Information) that were grouped into 6 groups as follows: myricetin derivatives (as the sum of myricetin 3-*O*-galactoside, 3-*O*-glucoside and 3-*O*-glucuronide derivatives), quercetin derivatives (as the sum of quercetin 3-*O*-galactoside, 3-*O*-glucoside, 3-*O*-glucuronide derivatives and quercetin aglycone), laritricin 3-*O*-glucoside, kaempferol derivatives (as the sum of kaempferol 3-*O*-galactoside, 3-*O*-glucoside, and 3-*O*-glucuronide derivatives), isorhamnetin 3-*O*-glucoside and syringetin 3-*O*-glucoside. Seven days after the addition of the MPs (P0), significantly lower levels of myricetin, quercetin, and kaempferol derivatives, as well as of total flavonol content, were observed in *A* wine compared to the control wine and to the other MP-treated wines (Figure 2A). This significant decrease in the total content of flavonols was also observed in *Z* and *B* wines, although to a lesser extent than in *A* wine.

After the cold treatment (P1), a decrease in the content of flavonols was observed in all wine samples compared to that of the previous sampling point analyzed (data not shown). This suggests that the application of low temperatures has a destabilizing effect on these compounds, promoting their precipitation. The fact that no significant differences were found in the total content of flavonols between the control wine and *Z* and *B* wines (Figure 2B) suggests that MP-Z and MP-B did not produce a significant stabilization of these compounds against cold, since the behavior of these wines was similar to that of the control wine. On the contrary, the destabilization of flavonols produced by cold seemed to be promoted by the addition of MP-A since a significant decrease in the content of myricetin, quercetin, and laritricin derivatives and in the total content of flavonols can be observed in *A* wine when compared to the other samples analyzed (Figure 2B).

After 45 days of storage at room temperature (P2), a significant decrease in the content of all flavonol derivatives was observed in *A* wine when compared to the control wine, being especially pronounced for quercetin and syringetin derivatives (∼18 and ∼15% reduction in their content, respectively) (Figure 2C). This decrease observed for all types of flavonols led to a significantly lower content of total flavonols in *A* wine compared with the control wine and to the other MP-enriched wines. In *Z* and *B* wines, the decrease in the total content of flavonols was smaller than the observed for *A* wine, although it was statistically significant when compared to the control wine. This suggests that the addition of MPs could favor the aggregation of flavonols, leading to wines with lower content of these phenolic compounds. These results agree with del Barrio-Galán et al., who observed lower concentrations of flavonol glycosides and aglycones in wines treated with a commercial inactive dry yeast preparation four months after the addition.^34^ On the contrary, Sartor et al. reported higher content of flavonols in rosé sparkling wines added with MPs after 12 months of aging on lees in comparison with the untreated wines.^33^ Alcalde-Eon et al. investigated the coaddition of seeds and MPs to a red wine and showed that MPs could partly revert the decrease in the content of flavonols caused by the addition of seeds after 6 months of treatment.^35^ The variable effects of MPs on wine flavonol content reported in the literature are in agreement with del Barrio-Galán et al., who showed variable effects in the content of flavonols after the addition of 5 commercial yeast derivative products to a red wine.^18^ This variability in the effects of MPs is likely due to differences in their characteristics. In the work of del Barrio-Galán et al., the wines added with two commercial yeast derivatives containing an important percentage of low MW polysaccharides (<11.8 kDa) showed higher contents of flavonols 8 weeks after treatment.^18^ However, this was not observed in the present study, because MP-A contained the highest percentage (∼54%) of low molecular weight polysaccharides (<27 kDa) (Table S1 of the Supporting Information) and was the MP extract that led to the wine with the lowest flavonol content.

It should be mentioned that the decrease in the content of flavonols was relevant, especially in the case of quercetin derivatives. All the MPs assayed led to wines with significantly lower contents of these compounds at the three analyzed sampling points. Moreover, a substantial decrease in the content of quercetin aglycone was observed, accounting for up to 54–60% in *A* wine at all three sampling points (data not shown). In *Z* and *B* wines, a noticeable decrease (40–45%) in the content of quercetin aglycone was also observed in P0 and P1. According to Terrier et al., the aggregation and precipitation of flavonols are restricted to aglycones, which exhibit lower solubility than their glycosides.^36^ Additionally, Xiao et al. studied the interaction between flavonols and bovine serum albumin through fluorescence quenching and found that glycosylation of flavonols in the C-ring reduced the affinity of the interaction.^37^ Therefore, the interaction of the MPs with flavonol glycosides may be less effective than their interaction with quercetin aglycone, resulting in lower adsorption of the glycosylated forms. This, coupled to the lower solubility of the flavonol aglycones, which would promote their aggregation and precipitation, could explain the great decrease observed in the content of quercetin aglycone.

Finally, it should be mentioned that the decrease in the content of flavonols observed in the MP-enriched wines could have a negative effect on the copigmented color of wines since flavonols have been described as the most efficient copigments. However, changes in the composition of other phenolic compounds, such as flavanols, should also be considered, as they can act as copigments of anthocyanins. These compounds use to be found in high amounts in wines, so, although they are less efficient copigments than flavonols, their role as copigments cannot be neglected.^2^ In this regard, the MP-enriched wines showed a higher content of flavanols in P2, as will be discussed later, which could counteract the possible loss of copigments due to the lesser content of flavonols.

#### Anthocyanins and Derived Pigment Content

**Figure 3:**
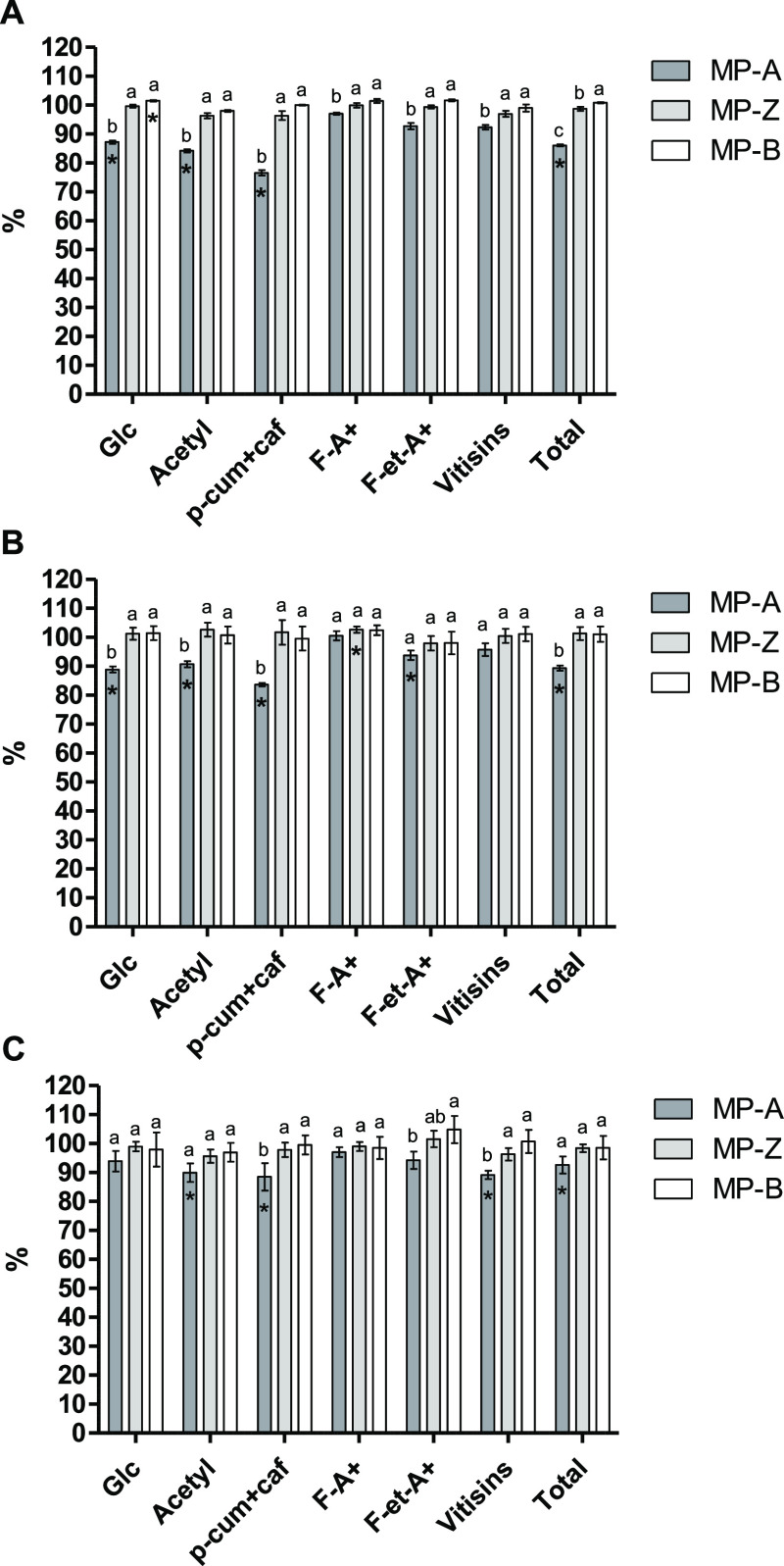
Percentage (%) of pigments with respect to the control wine at P0 (A), P1 (B), and P2 (C) sampling points. Different letters indicate significant differences among MP-supplemented wines (*p* < 0.05). Significant differences among the control wine and the wines added with MP are indicated with an asterisk (*p* < 0.05).

The analysis of wine samples by HPLC–DAD–ESI–MS allowed the identification of a total of 39 pigments (see Table S5 in the Supporting Information) that were grouped according to their structure in the following groups: anthocyanin glucosides (8 compounds, including the 5 anthocyanin monoglucosides and 3 diglucosides), acetylated anthocyanins (5 compounds), *p*-coumaroylated and caffeoylated anthocyanins (8 compounds), flavanol-anthocyanin direct condensation products (F-A^+^, 6 compounds), flavanol-anthocyanin acetaldehyde mediated condensation products (F-et-A^+^, 6 compounds), and vitisins (6 compounds). Total pigment content was calculated as the sum of all the above-mentioned compounds. Wine supplementation with MP-A led to a significantly lower content of total pigments than the control wine in the three sampling points analyzed (Figure 3A–C). Although this decrease was observed for all pigment families, it was more pronounced for grape native anthocyanins and, among these, for *p*-coumaroylated and caffeoylated anthocyanins. Some authors have observed that in wines treated with different yeast extracts containing MPs, a decrease in the content of anthocyanins was produced that was accompanied by an increase in the content of derived pigments. Consequently, these authors have hypothesized that MPs could favor the formation of anthocyanin-derived pigments.^13,14,18^ However, in our study, no significant increase in the content of derived pigments was found in *A* wine, suggesting an enhanced precipitation of anthocyanins caused by the addition of MP-A. These results agree with findings from other authors who describe the adsorption of anthocyanins on yeast MPs and polysaccharides leading to wines with lower concentrations of these compounds.^18,34^ In contrast, Guadalupe et al. found that the addition of commercial MPs to wine and the use of a MP-overproducing yeast strain had no impact on the content of monomeric anthocyanins.^8,9^ Moreover, del Barrio-Galán et al. did not observe the adsorption of anthocyanins on compounds released by lees or yeast derivatives (MPs, polysaccharides, etc.).^38^ Regarding the anthocyanin substitution in the C-ring, Morata et al. found that less polar anthocyanins (cinnamoyl derivatives) were more strongly adsorbed by the yeast cell wall than more polar acylated anthocyanins.^39^ In agreement with this, Gonçalves and co-workers reported that the interaction of MPs with coumaroylated anthocyanins was stronger than that with other anthocyanin derivatives, possibly due to their higher hydrophobicity.^40^ These results could indicate that the higher precipitation of *p*-coumaroylated and caffeoylated anthocyanins observed in *A* wine could be due to a higher propensity of MP-A to form hydrophobic interactions with wine pigments. In addition, MP-A showed an important content of ribose (∼22%) in its monosaccharide composition (Table S2 in the Supporting Information) that possibly comes from the nucleic acids present in this MP extract. Nucleic acids are negatively charged polyelectrolytes due to the presence of phosphate groups in their backbone.^41^ Anthocyanins are present in wine as different molecular forms in a pH-dependent equilibrium, whose predominant form in wine is the flavylium cation, which possesses a positive charge.^42^ Therefore, at wine pH (∼3.6), the negatively charged nucleic acids contained in MP-A could interact with the positively charged forms of anthocyanins, resulting in the formation of aggregates through ionic interactions that may eventually precipitate. This could partly explain why the addition of this MP-rich extract leads to a loss of pigments in *A* wine.

After wine stabilization by cold (P1), a significantly higher content of F-A^+^ can be found in *Z* wine compared to that in the control wine (Figure 3B), suggesting that MP-Z could slightly contribute to the colloidal stability of F-A^+^ compounds. The beneficial effect of MPs as colloidal stabilizers of wine coloring matter has been already described.^12,13^ However, after 45 days at room temperature (P2), the positive effect of MP-Z over the stability of F-A^+^ was no longer visible since there were no significant differences in the content of these pigments between *Z* wine and the control wine.

Regarding MP-B, it seems that this MP had a negligible effect in the content of wine pigments because the pigment profile of this wine did not differ considerably from that of the control wine in any of the sampling points analyzed.

The variability of the effects exerted by the three MPs on the wine pigment content is in good agreement with the controversial results reported in the literature. While some authors have reported that MPs could contribute positively to the chemical and colloidal stability of wine color,^13,14,35^ others found that anthocyanins and derived pigments can adsorb on MPs, leading to losses in the content of wine pigments and stable color.^21,34^ These variable effects of MPs addition could be linked to the different characteristics of these biopolymers, as aforementioned. In a previous study carried out in our laboratory, MPs were extracted from 4 yeast species by an ultrasound treatment and were assayed for the stabilization of red wine color. In this study, the MPs from *T. delbrueckii* were the ones that had impact on the colloidal and chemical stability of wine coloring matter.^13^ However, in the present study, only MP-Z seemed to slightly favor the colloidal stability of F-A^+^ pigments, pointing out that, besides the yeast of origin, the method of extraction of the MPs, which determines the structural and compositional characteristics of the MPs, highly conditions their techno-functional properties in wine.

#### Flavanol Content

**Figure 4:**
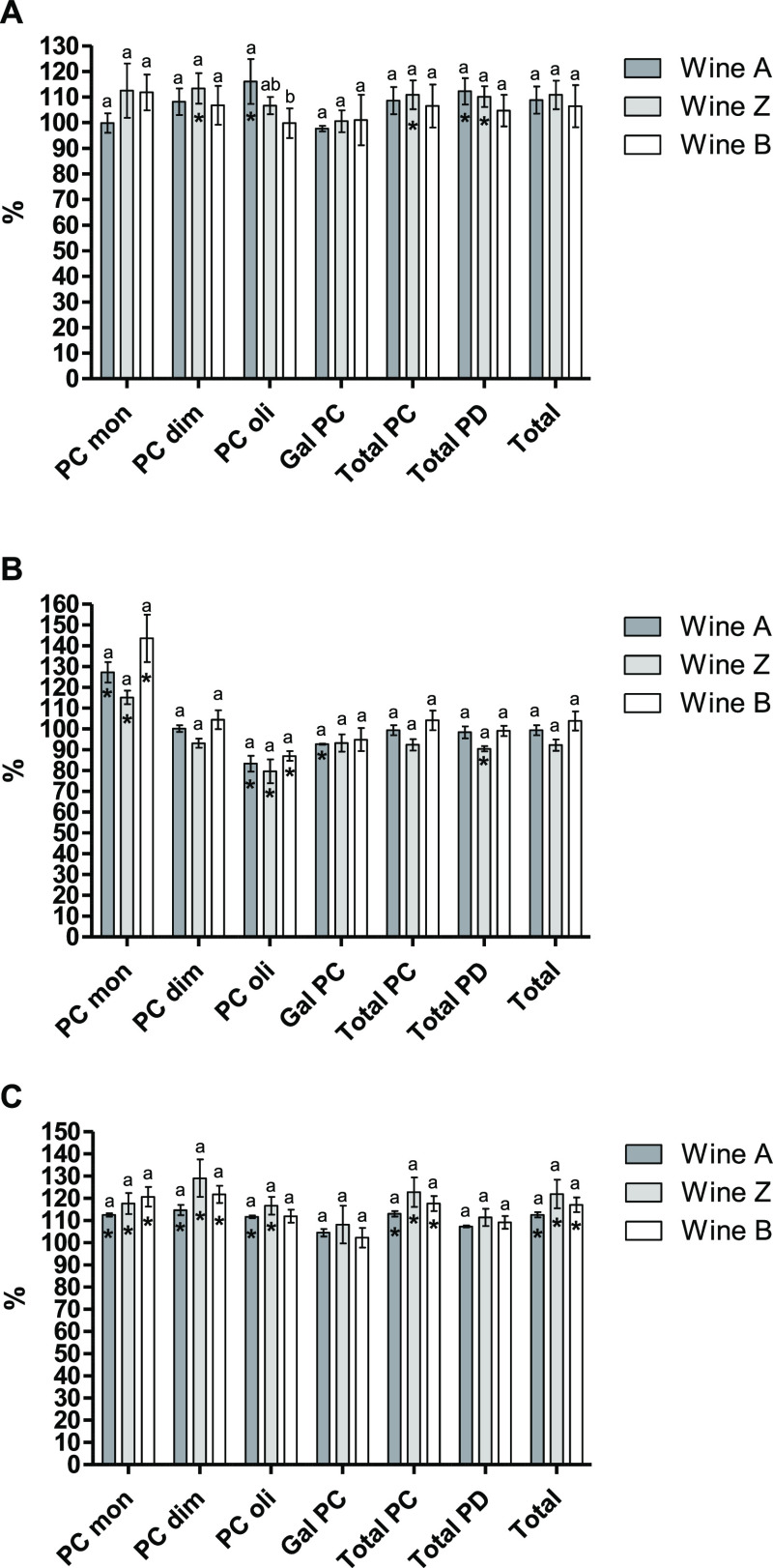
Percentage (%) of flavanols with respect to the control wine at P0 (A), P1 (B), and P2 (C) sampling points. PC mon: procyanidin monomers; PC dim: procyanidin dimers; PC oli: procyanidin oligomers; Gal PC: galloylated procyanidins; Total PC: total procyanidins; Total PD: total prodelphinidins. Different letters indicate significant differences among MP supplemented wines (*p* < 0.05). Significant differences among the control wine and the wines added with MP are indicated with an asterisk (*p* < 0.05).

HPLC–DAD–ESI–MS analyses of wine samples allowed the identification of 62 proanthocyanidins (PAs) (see Table S6 in the Supporting Information) that were grouped in PC monomers (2 compounds), PC dimers (6 compounds), PC oligomers (as the sum of 6 trimers, 9 tetramers, and 7 pentamers), galloylated PCs (5 compounds), and PDs (as the sum of 2 monomers, 11 dimers, and 14 trimers). Total flavanol content was calculated as the sum of the above-mentioned compounds. Seven days after the addition of the MPs (P0), *A* wine showed significantly higher levels of PC oligomers and of total PDs than the control wine. In addition, higher contents of PC dimers and total PCs can be found in *Z* wine compared to the control wine (Figure 4A). On the other hand, the application of cold led to a decrease in the content of PC oligomers in the MP-treated wines (Figure 4B). The addition of MPs could favor the aggregation of PC oligomers induced by the low temperatures, explaining the decrease in the content of these compounds. In the case of *A* wine, a significantly lower content of galloylated PCs than in the control wine can be observed too. Regarding *Z* wine, a decrease in the content of PDs was also produced. However, when it comes to PC monomers, significantly higher contents can be observed in the MP-enriched wines when compared to the control, suggesting that the addition of the MPs could enhance the stability against cold of these compounds. After 45 days of storage (P2), significantly higher contents of PC monomers and dimers were found in the wines supplemented with MPs (Figure 4C) compared to the control wine. Moreover, the content of PC oligomers was also significantly higher in *A* and *Z* wines than in the control wine. Given that PC monomers and, especially, PC dimers were the main type of flavanols present in wine, the higher content of these compounds translated into a higher content of PCs and total flavanols in the MP-supplemented wines. These results agree with del Barrio-Galán et al., who found that wines treated with different yeast derivatives enriched in MPs and polysaccharides showed higher levels of catechins and tannins than the control wines.^18^ On the contrary, Guadalupe and co-workers reported that the use of commercial MPs resulted in a decreased content of PAs, although no changes were produced in the content of monomeric flavanols.^21,22^ These controversial results again highlight the different effects exerted by MPs depending on their characteristics.

Two hypotheses can be proposed to explain this change in the flavanol content. On the one hand, MPs could act as colloidal stabilizers reducing flavanol aggregation and, consequently, resulting in an increased stability of PCs in solution. On the other hand, MPs could delay the polymerization reactions of flavanols that take place in wine under oxidative conditions, resulting in a higher content of these compounds. In fact, Rodrigues et al. reported that the addition of a commercial MP could influence the evolution of wine tannin aggregation, contributing to the delay of tannin polymerization reactions.^19^ Moreover, some authors have hypothesized that MPs could favor the formation of anthocyanin-derived pigments,^13,14,18^ suggesting that MPs could have an impact on the oxidative evolution of wine phenolic composition. In other words, it seems possible that MPs may influence not only the colloidal state of wine by modifying the aggregation of phenolic compounds but also the redox processes occurring in the wine matrix during oxidative aging. Further studies are needed to investigate the effect of MPs on the oxidative state of wine.

### Colorimetric Measurements

The color of wine samples was analyzed by UV–vis spectrophotometry in the three sampling points studied (P0, P1, and P2) and the CIELAB parameters were calculated. *A* wine showed a significantly higher luminosity (L*) than the other wines in the three sampling points analyzed (Table 1), indicating a loss of color intensity produced by the addition of MP-A, which correlates well with the decreased content of pigments of this wine. Moreover, color differences (ΔE*_ab_) between *A* wine and the control wine were higher than 3 in P0, P1, and P2 (Table 1) meaning that they were easily detectable by the human eye because it is generally considered that color differences higher than 3 can be visually detected.^43^ This loss of color produced by the adsorption of wine pigments in yeast MPs and polysaccharides has been already reported by other authors.^21,34^ It should be noted that a slight but significant decrease in the luminosity (L*) of *Z* wine was produced (Table 1), indicating that *Z* wine showed a higher color intensity than the control wine, which could be due to the higher content of F-A^+^ found in this wine, as has already been discussed. However, no substantial color modifications were produced upon the addition of MP-Z and MP-B since color differences with the control wine were not visible by the human eye (ΔE*_ab_ < 3) in any of the sampling points studied (Table 1).

**Table 1:** CIELAB Color Parameters of Wine Samples and Color Differences (ΔE*_ab_) between the Control and the MP-Enriched Wines Determined in the Three Sampling Points P0, P1, and P2^a^

	P0				P1				P2			
	L*	C*_ab_	*h*_*ab*_	ΔE*_ab_	L*	C*_ab_	*h*_*ab*_	ΔE*_ab_	L*	C*_ab_	*h*_*ab*_	ΔE*_ab_
control	60.7 ± 0.1 b	41.4 ± 0.05 a	–3.2 ± 0.05 a		61.7 ± 0.07 b	41.2 ± 0.03 a	–3.1 ± 0.03 a		57.5 ± 0.8 b	42.3 ± 0.1 a	–0.5 ± 0.1 ab	
*A* wine	62.5 ± 1.3 a	38.1 ± 0.1 b	–3.1 ± 0.1 a	3.8 ± 1.1	65.3 ± 0.06 a	37.3 ± 0.8 b	–3.5 ± 0.8 a	5.3 ± 0.3	61.9 ± 0.3 a	38.7 ± 0.4 b	–0.9 ± 0.4 b	5.7 ± 0.3
*Z* wine	60.7 ± 0.1 b	41.2 ± 0.1 a	–3.1 ± 0.05 a	0.2 ± 0.06	61.4 ± 0.07 c	41.0 ± 0.2 a	–2.8 ± 0.07 a	0.7 ± 0.04	57.6 ± 0.3 b	42.4 ± 0.2 a	–0.5 ± 0.1 ab	0.3 ± 0.1
*B* wine	60.7 ± 0.1 b	41.1 ± 0.1 a	–3.1 ± 0.06 a	0.3 ± 0.08	61.6 ± 0.1 bc	40.9 ± 0.1 a	–2.9 ± 0.1 a	0.6 ± 0.08	57.3 ± 0.4 b	42.5 ± 0.2 a	–0.3 ± 0.1 a	0.5 ± 0.2

a Different letters within each column indicate significant differences (*p* < 0.05).

### Sensory Analysis

The sensory analysis conducted after 45 days of storage of the wine samples (P2) revealed that the intensity of astringency was lower for *A* and *Z* wines than for control and *B* wines (Figure S1 in the Supporting Information), suggesting that MP-A and MP-Z were able to reduce wine astringency. This is in agreement with previous works that have demonstrated the ability of some MPs to modulate wine astringency.^11,14,15^ It should be noted that this decrease in wine astringency was not related to a fining effect of MPs, since the content of total flavanols in *A* and *Z* wines were, respectively, around 13 and 22% higher than that in the control wine (Figure 4C). Flavanols are generally considered the main phenolic compounds responsible for wine astringency^4^ and some studies point out that there is a direct correlation between tannin concentration and perceived astringency.^44^ However, *A* and *Z* wines, which were less astringent, showed a significantly higher content of flavanols than the control wine. Thus, changes in the profile of other phenolic compounds besides flavanols should also be considered as they may have an impact on the astringency of the wines. In this regard, the addition of MP-A and MP-Z led to an increased content of flavanols and, at the same time, significantly reduced the content of flavonols and, in the case of MP-A, also of wine pigments. Given that some studies describe flavonols as astringent compounds,^45,46^ the decrease in the content of these compounds in *A* and *Z* wines could contribute to explaining the decrease in wine astringency. Furthermore, Ferrero-del-Teso et al. found that anthocyanins and dimeric flavanol-anthocyanin condensates can be related to the astringent sensation^47^ and Ferrer-Gallego et al. have demonstrated the ability of anthocyanins to interact with salivary proteins.^48^ Consequently, the decrease in the astringency intensity of *A* wine could also be due to its lower content of pigments.

However, the fact that *B* wine, like *A* and *Z* wines, showed higher contents of flavanols and lower contents of flavonols than the control wine but its astringency intensity did not differ significantly from the latter suggests that considering changes in the phenolic composition caused by the presence of MPs may not be sufficient to explain the decrease in wine astringency observed for *A* and *Z* wines. Therefore, it is important to consider other factors such as the formation of stable aggregates between MPs and phenolic compounds in the solution. These aggregates may modify the interaction between phenolic compounds and salivary proteins, and it could explain the observed effects. The change in the aggregation state of tannins influencing astringency caused by the presence in the wine matrix of biopolymers (like proteins and polysaccharides) is a hypothesis already proposed by other authors.^49^ In this regard, some authors have demonstrated the ability of MPs to interact with phenolic compounds, resulting in the formation of MP-phenolic compound complexes that lead to the modification of the interaction between phenolic compounds and salivary proteins.^11,30,50^ However, the characteristics of the MPs seem to condition MP-phenolic compound-salivary protein interactions as well as the type of aggregates formed. In this sense, Manjón et al. proposed that a larger protein fraction in the MP increases the possibility of interaction with flavanols, increasing its ability to bind more flavanol molecules. Furthermore, there could also exist a relationship between the size of the MP and its potential to modulate astringency.^11^ Following this line, Wang and co-workers reported that MPs with high protein content and high MW appear to have more interaction sites to bind to other wine components and a stronger ability to bind phenolic compounds and proteins.^15^ MP-A, MP-Z, and MP-B showed similar protein percentages, but their average MW differed significantly (Table S1 in the Supporting Information). The fact that MP-A and MP-Z were larger biomolecules than MP-B could indicate that these two MPs may have a higher number of binding sites and, consequently, could bind a higher number of phenolic molecules, preventing their interaction with salivary proteins, which would lead to a decrease in wine astringency intensity. However, further studies are needed in order to determine the formation of MP-phenolic compound aggregates and their characteristics. In addition, the impact of the formation of these aggregates in the interaction between phenolic compounds and salivary proteins should also be analyzed in order to characterize the molecular mechanisms of the astringency modulatory effect of these MPs.

In conclusion, the three MP extracts obtained from *T. delbrueckii* modified the phenolic composition of red wine in different ways depending on their structure and composition. This highlights the impact of the extraction method on the techno-functional properties of MPs. The addition of MP-A and MP-Z resulted in a reduction of the perceived wine astringency. This decrease in wine astringency seemed not to be related to a reduction in the content of main astringent molecules, as the MP-treated wines showed an increased content of flavanols. Therefore, besides analyzing the changes in the phenolic content upon MP addition, the clarification of the mechanism of phenolic compound-polysaccharide complex formation seems to be essential for understanding how changes in the aggregation state can influence the perceived astringency. Our results suggest that the astringent modulatory effect of MP-A and MP-Z could be linked to their higher MW, allowing them to bind more astringent molecules and consequently reducing their reactivity toward salivary proteins. The negative effect of MP-A on wine color cannot be neglected and emphasizes the importance of evaluating together the effects of MPs on wine sensory properties related to phenolic compounds in order to avoid undesired side effects. Among the three MPs tested, MP-Z showed the most favorable impact, since its addition resulted in a decrease of wine astringency without affecting wine color. This finding could offer valuable insights for the industry in obtaining MPs with similar characteristics to MP-Z.

## Supporting Information Available

Molecular weight (MW) distribution, average MW (kDa), and total protein content (*w/w*) of the MP extracts; MP-A: MP extract obtained by induced autolysis; MP-Z: MP extract obtained by enzymatic extraction; MP-B: MP extract obtained by alkaline extraction; different letters within each column indicate significant differences (*p* < 0.05); monosaccharide composition of the MP extracts. Man: mannose; Glc: glucose; Rib: ribose; Xyl: xylose; MP-A: MP extract obtained by induced autolysis; MP-Z: MP extract obtained by enzymatic extraction; MP-B: MP extract obtained by alkaline extraction; different letters within each row indicate significant differences (*p* < 0.05); main phenolic acids identified in the wine samples; main flavonols identified in the wine samples; main anthocyanins and anthocyanin-derived pigments identified in the wine samples; Dp: delphinidin; Cy: cyanidin; Pt: petunidin; Pn: peonidin; Mv: malvidin; glc: glucoside; p coum: *p*-coumaroyl; (E)C: (epi)catechin; GC: gallocatechin; F-A+: flavanol anthocyanin direct condensation products; F-et-A+: flavanol anthocyanin acetaldehyde mediated condensation products; Vit: vitisin. Asterisks in F-et-A+ are used to identify the isomers (although the type of isomerism is unknown); main flavanols identified in the wine samples. PC: procyanidin; PD: prodelphinidin; a* and b* coordinates of the CIELAB color space determined for the wine samples the three sampling points P0, P1 and P2; and stringency intensity of the wine samples rated in the LMS (PDF)

## Abbreviations

MP: mannoprotein
MW: molecular weight
HCAs: hydroxycinnamic acids
HBAs: hydroxybenzoic acids
LMS: labeled magnitude scale
F-A^+^: flavanol-anthocyanin direct condensation products
F-et-A^+^: flavanol-anthocyanin acetaldehyde mediated condensation products
PA: proanthocynidin
PC: procyanidin
PD: prodelphinidin
