# Supplementary material for: Potential Use of Torulaspora delbrueckii As a New Source of Mannoproteins of Oenological Interest
Source: J Agric Food Chem. 2024 May 9;72(20):11606–16. doi: 10.1021/acs.jafc.4c01001 (PMC11117404; doi:10.1021/acs.jafc.4c01001)

SUPPORTING INFORMATION

Potential use of *Torulaspora delbrueckii* as a new source of mannoproteins of oenological interest

Oyón-Ardoiz, María[1]; Manjón, Elvira*[1]; Escribano-Bailón, María Teresa[1]; García-Estévez, Ignacio[1]

[1] Department of Analytical Chemistry, Nutrition and Food Science, Universidad de Salamanca, Salamanca, E37007, Spain

*Corresponding author: Manjón, Elvira

E-mail address: elvira87@usal.es

Table S1. Molecular weight (MW) distribution, average MW (kDa) and total protein content (*w/w*) of the MP extracts. MP-A: MP extract obtained by induced autolysis; MP-Z: MP extract obtained by enzymatic extraction; MP-B: MP extract obtained by alkaline extraction. Different letters within each column indicate significant differences ($p < 0.05$).

	MW (kDa)	%	Average MW (kDa)	Protein content (%)
MP-A	902-171	27.7	129±2 a	3.4±0.3 a
	171-58	7.7		
	58-27	11.0		
	27-2	53.6		
MP-Z	481-190	16.3	116±2 b	3.5±0.2 a
	190-35	70.1		
	35-4	13.6		
MP-B	376-11	94.5	94±5 c	4.2±0.6 a
	11-3	5.5		

Table S2. Monosaccharide composition of the MP extracts. Man: mannose; Glc: glucose; Rib: ribose; Xyl: xylose. MP-A: MP extract obtained by induced autolysis; MP-Z: MP extract obtained by enzymatic extraction; MP-B: MP extract obtained by alkaline extraction. Different letters within each row indicate significant differences ($p < 0.05$).

	MP-A	MP-Z	MP-B
Man (%)	74.6±0.4 c	86.3±0.2 b	96.0±0.1 a
Glc (%)	2.2±0.2 c	12.5±0.2 a	3.0±0.04 b
Rib (%)	22.1±0.3 a	0.2±0.1 b	-
Xyl (%)	1.1±0.1 a	1.1±0.2 a	1.0±0.1 a

Table S3. Main phenolic acids identified in the wine samples.

Hydroxycinnamic acids (HCA)	
t_R (min)	Compound identity
12.8	*Cis*-caftaric acid
14.2	*Trans*-caftaric acid
18.2	*Cis*-cutaric acid
19.6	*Trans*-cutaric acid
22.9	*Trans*-fertaric acid
23.9	Caffeic acid
36.8	*p*-coumaric acid
Hydroxybenzoic acids (HBA)	
9.2	Gallic acid

Table S4. Main flavonols identified in the wine samples.

Myricetin derivatives	
t_R (min)	Compound identity
33.0	Myricetin 3-*O*-galactoside
33.9	Myricetin 3-*O*-glucoside + Myricetin 3-*O*-glucuronide
Quercetin derivatives	
39.6	Quercetin 3-*O*-galactoside
40.3	Quercetin 3-*O*-glucoside + Quercetin 3-*O*-glucuronide
52.6	Quercetin aglycone
Laritricin derivatives	
41.0	Laritricin 3-*O*-glucoside
Kaempferol derivatives	
42.7	Kaempferol 3-*O*-galactoside
44.0	Kaempferol 3-*O*-glucoside + Kaempferol 3-*O*-glucuronide
Isorhamnetin derivatives	
44.9	Isorhamnetin 3-*O*-glucoside
Syringetin derivatives	
45.0	Syringetin 3-*O*-glucoside

Table S5. Main anthocyanins and anthocyanin-derived pigments identified in the wine samples. Dp: delphinidin; Cy: cyanidin; Pt: petunidin; Pn: peonidin; Mv: malvidin; glc: glucoside; *p* coum: *p*-coumaroyl; (E)C: (epi)catechin; GC: gallocatechin; F-A+: flavanol anthocyanin direct condensation products; F-et-A+: flavanol anthocyanin acetaldehyde mediated condensation products; Vit: vitisin. Asterisks in F-et-A+ are used to identify the isomers (although the type of isomerism is unknown).

Anthocyanin glucosides	
t_R (min)	Compound identity
19.9	Dp-diglc
22.8	Dp-3-glc
26.0	Pt-diglc
27.0	Cy-3-glc
29.8	Pt-3-glc
33.5	Mv-diglc
35.4	Pn-3-glc
37.0	Mv-3-glc
Acetylated anthocyanins	
39.7	Dp-3-acetylglc
42.4	Cy-3-acetylglc
43.0	Pt-3-acetylglc
45.4	Pn-3-acetylglc
45.8	Mv-3-acetylglc
p-coumaroylated and caffeoylated anthocyanins	
44.1	Dp-3-*p* coumglc
46.6	Pt-3-*p* coumglc *cis*
47.4	Mv-3-cafglc
47.8	Cy-3-*p* coumglc
48.2	Pt-3-*p* coumglc *trans*
48.9	Mv-3-*p* coumglc *cis*
50.4	Pn-3-*p* coumglc
50.5	Mv-3-*p* coumglc *trans*

Continuation of Table S5.

F-A+	
t_R (min)	Compound identity
5.7	Dp-3-glc-GC
7.5	Pt-3-glc-GC
11.0	Mv-3-glc-GC
16.6	Pt-3-glc-C
20.4	Pn-3-glc-EC
21.5	Mv-3-glc-C
F-et-A+	
41.0	Pt-3-glc-etil-C
42.3	Mv-3-glc-etil-EC*
42.6	Mv-3-glc-etil-C*
43.7	Mv-3-glc-etil-C*
44.5	Mv-3-glc-etil-EC*
49.3	Mv-3-*p* coumglc-etil-C
Vitisins	
21.7	Vit A Dp-glc
37.7	Vit A Mv-glc
40.2	Vit A Mv-3-acetylglc
40.8	Vit B Mv-3-glc
52.7	Vit vinylphenol + Mv-3-glc
54.9	Vit vinylphenol + Mv-3-acetylglc

Table S6. Main flavanols identified in the wine samples. PC: procyanidin; PD: prodelphinidin.

PC monomers	
t_R (min)	Compound identity
19.5	Catechin
29.2	Epicatechin
PC dimers	
15.5	Dimer B1
17.5	Dimer B3
22.5	Dimer B4 + dimer B6
24.6	Dimer B2
39.6	Dimer B7
PC trimers	
10.2	Trimer 1
18.5	Trimer C2
20.2	Trimer 2
20.9	Trimer 3
34.5	Trimer 4
36.4	Trimer C1
PC tetramers	
14.1	Tetramer 1
15.9	Tetramer 2
17.9	Tetramer 3
21.0	Tetramer 4
26.6	Tetramer 5
27.7	Tetramer 6
32.5	Tetramer 7
34.2	Tetramer 8
41.3	Tetramer 9
PC pentamers	
14.5	Pentamer 1
20.7	Pentamer 2
23.3	Pentamer 3
25.7	Pentamer 4
38.5	Pentamer 5
43.3	Pentamer 6
44.8	Pentamer 7

Continuation of Table S6.

Galloylated PCs	
t_R (min)	Compound identity
31.5	Galloylated dimer 1
32.8	Galloylated dimer 2
36.2	Galloylated dimer 3
39.9	Galloylated dimer 4
40.1	Galloylated dimer 5
PD monomers	
11.8	Gallocatechin
16.6	Epigallocatechin
PD dimers	
9.1	Dimer 1
10.4	Dimer 2
10.6	Dimer 3
11.7	Dimer 4
13.3	Dimer 5
13.8	Dimer 6
15.3	Dimer 7
16.7	Dimer 8
17.8	Dimer 9
22.9	Dimer 10
25.1	Dimer 11
PD trimers	
8.8	Trimer 1
9.2	Trimer 2
9.5	Trimer 3
11.2	Trimer 4
12.5	Trimer 5
13.2	Trimer 6
13.9	Trimer 7
14.7	Trimer 8
14.9	Trimer 9
15.2	Trimer 10
16.7	Trimer 11
17.7	Trimer 12
22.9	Trimer 13
30.3	Trimer 14

Table S7. a* and b* coordinates of the CIELAB color space determined for the wine samples in the three sampling points P0, P1 and P2.

	P0		P1		P2	
	a*	b*	a*	b*	a*	b*
Control	41.3 ± 0.2	-2.3 ± 0.2	41.1 ± 0.06	-2.4 ± 0.03	42.3 ± 0.7	-0.3 ± 0.1
A wine	38.1 ± 0.5	-2.0 ± 0.05	37.3 ± 0.5	-2.3 ± 0.5	38.7 ± 0.1	-0.6 ± 0.3
Z wine	41.2 ± 0.1	-2.3 ± 0.04	41.0 ± 0.2	-2.0 ± 0.05	42.4 ± 0.2	-0.4 ± 0.08
B wine	41.1 ± 0.1	-2.2 ± 0.04	40.9 ± 0.1	-2.1 ± 0.08	42.5 ± 0.2	-0.3 ± 0.06

Figure S1. Astringency intensity of the wine samples rated in the Labeled Magnitude Scale.

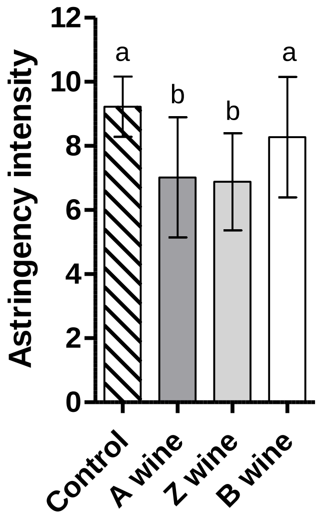

Supplement: Supplementary file 1 — jf4c01001_si_001.pdf [file jf4c01001_si_001.pdf]
